# *In vivo* role of checkpoint kinase 2 in signaling telomere dysfunction

**DOI:** 10.1111/acel.12237

**Published:** 2014-06-12

**Authors:** María García-Beccaria, Paula Martínez, Juana M Flores, Maria A Blasco

**Affiliations:** 1 Molecular Oncology Program, Telomeres and Telomerase Group, Spanish National Cancer Research Centre (CNIO)Madrid, E-28029, Spain; 2 Animal Surgery and Medicine Department, Faculty of Veterinary Science, Complutense University of MadridMadrid, E-28040, Spain

**Keywords:** aging, DNA damage, genetics, lifespan, telomerase, telomere

## Abstract

Checkpoint kinase 2 (CHK2) is a downstream effector of the DNA damage response (DDR). Dysfunctional telomeres, either owing to critical shortening or disruption of the shelterin complex, activate a DDR, which eventually results in cell cycle arrest, senescence and/or apoptosis. Successive generations of telomerase-deficient (*Terc*) mice show accelerated aging and shorter lifespan due to tissue atrophy and impaired organ regeneration associated to progressive telomere shortening. In contrast, mice deficient for the shelterin component TRF1 in stratified epithelia show a rapid and massive induction of DDR, leading to perinatal lethality and severe skin defects. In both mouse models, p53 deficiency can rescue survival. Here, we set to address the role of CHK2 in signaling telomere dysfunction in both mouse models. To this end, we generated mice doubly deficient for *Chk2* and either *Terc* (*Chk2*^*−/−*^ *Terc*^*−/−*^*)* or *Trf1* (*Trf1*^*Δ/Δ*^ *K5Cre Chk2*^*−/−*^). We show that *Chk2* deletion improves *Terc*-associated phenotypes, including lifespan and age-associated pathologies. Similarly, *Chk2* deficiency partially rescues perinatal mortality and attenuates degenerative pathologies of *Trf1*^*Δ/Δ*^ *K5Cre* mice. In both cases, we show that the effects are mediated by a significant attenuation of p53/p21 signaling pathway. Our results represent the first demonstration of a role for CHK2 in the *in vivo* signaling of dysfunctional telomeres.

## Introduction

Telomere dysfunction is caused by either critical telomere shortening or loss of the protein complex that protects telomeres, the so-called shelterin (de Lange, 2005a). Dysfunctional telomeres behave as double strand breaks (DSBs) and trigger a persistent DNA damage response (DDR) (d’Adda di Fagagna *et al*., 2003; de Lange, 2005a). DSBs are among the most deleterious lesions that challenge genomic integrity. Concomitant to the repair of DSBs, a rapid signaling cascade must be coordinated at the lesion site to prevent cell division by activation of cell cycle checkpoints. Ataxia telangiectasia mutated (ATM) and ATM and Rad-3-related (ATR) protein kinases are among the earliest signaling molecules known to initiate the transduction cascade at damage sites. This cascade ultimately results in the activation of p21 and p53, leading to senescence/apoptosis (Takai *et al*., 2003; von Zglinicki *et al*., 2005). ATM has an essential role in signaling from DSBs arising from ionizing radiation (IR) through a CHK2-dependent pathway, while ATR is typically involved in signaling from replication-linked SSBs through the CHK1 kinase. Recent findings, however, have demonstrated an active cross talk between ATM and ATR signaling pathways in response to DNA damage (Matsuoka *et al*., 2000; Murga *et al*., 2009). Both CHK1 and CHK2 can activate p53 upon DNA damage (Shieh *et al*., 2000) and are important for cell cycle checkpoints, however, while CHK1 is crucial for intra-S and G2/S transition, CHK2 is more important for G1/S checkpoint (Liu *et al*., 2000; Takai *et al*., 2000, 2002; Hirao *et al*., 2002).

Telomere shortening is one of the molecular pathways underlying organismal aging (Lopez-Otin *et al*., 2013). Telomerase-deficient mice show progressive telomere shortening, eventually impairing tissue regeneration and leading to tissue atrophy and premature aging phenotypes, as well as to cancer resistance (Greenberg *et al*., 1998; Lee *et al*., 1998; Rudolph *et al*., 1999; Gonzalez-Suarez *et al*., 2000). p53 deficiency can rescue both survival and tumorigenesis in *Terc*^*−/−*^ *p53*^*−/−*^ mice, indicating that p53 is one of the main downstream mediators of the cellular response to DDR triggered by short telomeres (Artandi *et al*., 2000).

TRF1 is one of the six components of shelterin, which has an essential role in protecting telomeres from fusions and from telomere fragility resulting from telomere replication (De Lange, 2005b). Abrogation of *Trf1* in mice results in early embryonic lethality (Karlseder *et al*., 2003). Conditional deletion of *Trf1* in mouse stratified epithelia leads to perinatal lethality and severe skin morphogenesis defects, which are concomitant with rapid induction of telomere-originated DNA damage and activation of the p53/p21 pathway (Martinez *et al*., 2009). Similar to that shown for *Terc*-deficient mice, deletion of *p5*3 in *Trf1*^*Δ/Δ*^ *K5Cre* mice rescues survival and leads to increased cancer incidence, indicating that p53 is also a main mediator of the cellular response to DDR induced by uncapped telomeres (Martinez *et al*., 2009).

Interestingly, it has been recently shown that deletion of *Trf1* induces phosphorylation of both the ATR and ATM downstream kinases CHK1 and CHK2, respectively, suggesting that both checkpoint kinases may be important to mediate telomere dysfunction owing to telomere uncapping (Martinez *et al*., 2009; Sfeir *et al*., 2009). In line with this, inhibition of either ATM or ATR kinases in *Trf1*-deficient MEFs can significantly rescue DNA damage *in vitro* (Martinez *et al*., 2009). However, whether attenuation of these pathways rescues *Trf1* deficiency-associated phenotypes *in vivo* is not known.

Several studies have previously addressed the role of CHK2 in the response to dysfunctional telomeres *in vitro* cultured human cells (Thanasoula *et al*., 2012; Cesare *et al*., 2013). Thus, Cesare *et al*. (2013) showed that telomere deprotection resuting from TRF2 downregulation to levels that do not induce telomere–telomere fusions, results in p53-mediated G1 cell cycle arrest independently of CHK2 phosphorylation. Only when TRF2 was fully abrogated leading to chromosome fusions, the authors observed CHK2 phosphorylation (Cesare *et al*., 2013). The authors concluded that very few TRF2 molecules at telomeres are sufficient to prevent the nonhomologous end joining pathway (NHEJ) and to inhibit CHK2 activation, thereby allowing the cells to continue through mitosis avoiding the G2/M arrest (Cesare *et al*., 2013). In contrast, Thanasoula *et al*. (2012) showed that TRF2 depletion leads to CHK2 phosphorylation and to G2/M arrest, preventing fusions and genome instability.

In the case of the mouse, we have previously shown that dysfunctional telomeres induced by *Trf1* deletion lead to Chk2 phosphorylation and G2/M arrest (Martinez *et al*., 2009). Furthermore, abolishment of end-to-end fusions owing to Trf1 deficiency by simultaneously deleting 53 bp1, and essential component of the NHEJ, does not inhibit Chk2 phosphorylation or ‘*in vivo*’ G2/M arrest (Martinez *et al*., 2009). Similarly, mouse cells depleted for Trf2 and lacking ligase 4, another essential component of the NHEJ, do not show telomere–telomere fusions but show Chk2 phosphorylation (Celli & de Lange, 2005). Thus, in contrast to human cells, mouse telomere dysfunction induced by either depleting TRF1 or TRF2 leads to Chk2 phosphorylation independently of chromosome fusions.

Here, we set to address the role of CHK2 activation in response to dysfunctional telomeres *in vivo*. In particular, we set to address whether *Chk2* deficiency could rescue phenotypes associated to telomere dysfunction in *Terc* and *Trf1* deficient mouse models previously generated by us (Blasco *et al*., 1997; Martinez *et al*., 2009).

*Chk2*-deficient mice are viable and do not show increased cancer incidence (Donehower *et al*., 1992; Barlow *et al*., 1996; Hirao *et al*., 2002), constituting an interesting model to test the importance of this checkpoint kinase in mediating telomere dysfunction *in vivo*. Thus, here, we generated *Trf1*^*Δ/Δ*^ *K5Cre Chk2*^*−/−*^ and *Chk2*^*−/−*^ *Terc*^*−/−*^ compound mice. The findings described here indicate that *Chk2* abrogation can partially rescue both survival and some of the phenotypes associated to both *Terc* and *Trf1* deficiencies, revealing a potential for Chk2 inhibition in ameliorating phenotypes associated to both critically short telomeres and severe telomere dysfunction.

## Results

### *Chk2* deficiency rescues mouse survival and degenerative pathologies in *Trf1*-deficient mice

**Figure 1:**
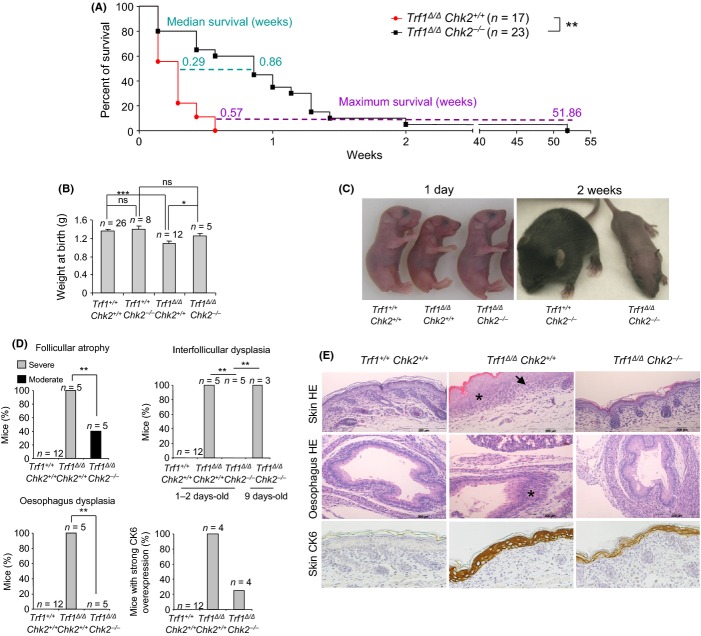
*Chk2* deficiency partially rescues *Trf1*^*Δ/Δ*^ *K5Cre* associated phenotypes and lethality (A) Survival curve of *Trf1*^*Δ/Δ*^ *K5Cre Chk2*^*−/−*^ and *Trf1*^*Δ/Δ*^ *K5Cre Chk2*^*+/+*^ mice. Statistical analysis was done by the Log-rank (Mantel–Cox) test. (B) Weight at birth of the indicated genotypes. (C) Representative images of newborns and 2-week-old mice of the indicated genotypes. (D) Percentage of newborns that show severe or moderate skin follicular atrophy, skin interfollicular dysplasia, esophagus dysplasia, and strong CK6 expression. Chi-square test was performed for statistical analysis. Severe follicular atrophy is characterized by a reduced number of hair follicle primordial that are completely undeveloped. Moderate follicular atrophy is characterized by a decreased differentiation degree as compared to wild-type. Interfollicular dysplasia is characterized by undifferentiated keratinocytes, cellular depolarization and hyperkeratosis. (E) Representative images of hematoxylin–eosin and cytokeratin 6 staining in newborn skin and esophagus of the indicated genotypes. Dysplasic areas are marked with an asterisk and undeveloped hair follicles with an arrow. *P < 0.05; **P < 0.01; ***P < 0.001.

To address the *in vivo* role of CHK2 in signaling telomere dysfunction owing to *Trf1* deficiency, we crossed *Trf1*^*Δ/Δ*^ *K5Cre* mice (Martinez *et al*., 2009) with mice with whole-body *Chk2* deletion, *Chk2*^*−/−*^ (Hirao *et al*., 2002). Interestingly, *Chk2* deficiency significantly rescued the survival of *Trf1*^*Δ/Δ*^ *K5Cre* mice, with one doubly deficient mice surviving for as long as 1 year of age compared to a maximum survival of 4 days for single *Trf1*-deficient littermates (Fig. 1A). On average, the median survival of the *Trf1*^*Δ/Δ*^ *K5Cre Chk2*^*−/−*^ mice was threefold higher than the *Trf1*^*Δ/Δ*^ *K5Cre* mice, 0.9 and 0.3 weeks, respectively (Fig. 1A). In agreement with the increased survival, we also observed a significant rescue of body weight in *Trf1*^*Δ/Δ*^ *K5Cre Chk2*^*−/−*^ newborns compared to the *Trf1*^*Δ/Δ*^ *K5Cre* newborns (Fig. 1B–C).

**Figure 2:**
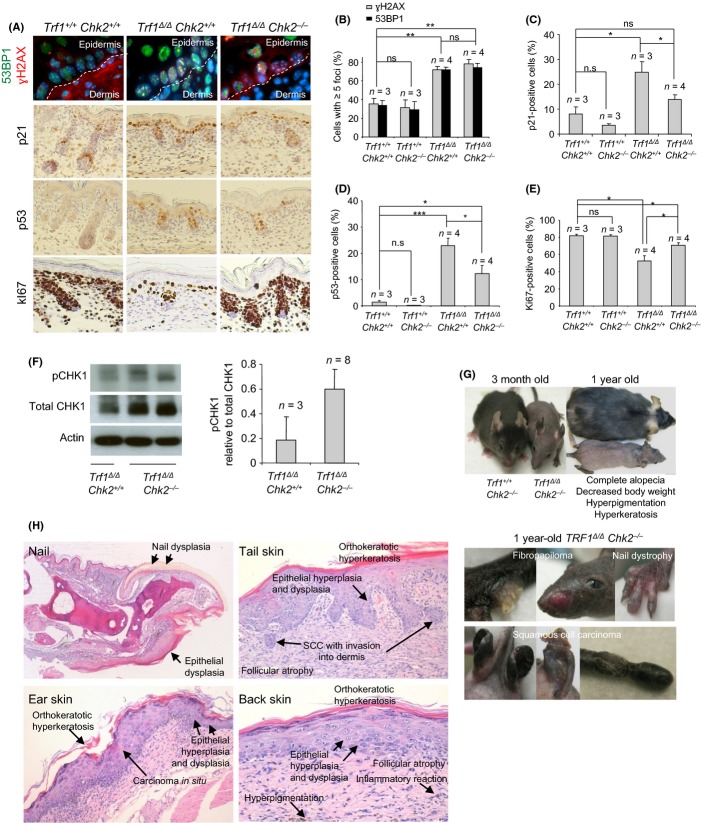
*Chk2* deficiency increases epithelial tumor incidence in *Trf1*^*Δ/Δ*^ *K5Cre* mice (A) Representative images of γH2AX and 53BP1 double immunofluorescence, and of p21, p53 and Ki67 immunohistochemistries performed on newborn skin. (B–E) Percentage of cells showing 5 or more γH2AX and 53BP1 damage foci (B), p21 positive cells (C), p53 positive (D) and Ki67 positive cells (E) in the newborn basal skin layer of the indicated genotypes. Of 1500–2000 cells were analyzed per genotype. *T* test was performed for statistical analysis. *P < 0.05; **P < 0.01; ***P < 0.001. Error bars represent standard error. *n*, number of mice analyzed per genotype. (F) Representative image and quantification by western blot of phospho-CHK1 normalized to total CHK1 in newborn keratinocytes. *n* represents the number of mice analyzed per genotype. (G–H) Representative images of the longer-lived *TRF1*^*Δ/Δ*^ *K5Cre Chk2*^*−/−*^ mouse compared to a wild-type littermate at different ages. Macroscopic lesions, fibropapiloma, nail dystrophy, and squamous cell carcinoma in ear and tail skin are shown.

In an analogous manner, the frequency and severity of epithelial pathologies associated to *Trf1* deficiency such as hair follicle atrophy, skin interfollicular dysplasia and esophagus dysplasia were also significantly rescued in *Trf1*^*Δ/Δ*^ *K5Cre Chk2*^*−/−*^ newborns (1–2 day old mice) compared to the *Trf1*^*Δ/Δ*^ *K5Cre Chk2*^*+/+*^ age-matched controls (Fig 1D,E). Aberrant overexpression of cytokeratin 6 associated to *Trf1* deficiency was also rescued in the *Chk2*-deficient background (Fig. 1D,E). Interestingly, at 9 days of age, at which point all single mutant *Trf1*^*Δ/Δ*^ *K5Cre* mice were dead, surviving *Trf1*^*Δ/Δ*^ *K5Cre Chk2*^*−/−*^ mice showed extensive dysplasia in the skin, suggesting an increased preneoplastic growth associated to massive *Trf1*-induced DNA damage in the absence of *Chk2* (Fig. 1D; see also Fig. 2).

### *Chk2* deficiency reduces p53 induction and increases proliferation of the skin of *K5Cre Trf1*^*Δ/Δ*^ *Chk2*^*−/−*^ newborns

To further investigate how *Chk2* deficiency could rescue both the severity of the skin pathologies and survival of *Trf1*^*Δ/Δ*^ *K5Cre Chk2*^*−/−*^ mice compared to *Trf1*^*Δ/Δ*^ *K5Cre* littermates, we first set to quantify DNA damage in the skin of these mice by determining the number of cells showing DNA damage foci as indicated by staining with γH2Ax and 53BP1. Both *Trf1*^*Δ/Δ*^ *K5Cre Chk2*^*+/+*^ and *Trf1*^*Δ/Δ*^ *K5Cre Chk2*^*−/−*^ newborn skin showed approximately 80% of the basal cells positive for γH2Ax and 53BP1 staining (>5 foci per cell), indicating similarly high amounts of DNA damage in both cases, in agreement with similarly high levels of telomere uncapping owing to TRF1 deficiency (Fig. 2A–B). Conditional Trf1deletion in MEFs by adenoviral infection with the Cre recombinase induces Chk2 phosphorylation (Fig. S1A,B) (Martinez *et al*., 2009). We also found similar amounts of DNA damage, as well as similar frequencies of chromosomal aberrations associated to *Trf1* deficiency in *in vitro* grown MEFs deficient for both *Trf1* and *Chk2* (Fig. S1), thus confirming the *in vivo* findings.

Interestingly, although the amount of DNA damage was similar independently of the *Chk2* status, we found a decreased induction of p21 and p53 in the epidermis of *Trf1*^*Δ/Δ*^ *K5Cre Chk2*^*−/−*^ doubly deficient mice compared to that of single *Trf1* knockouts (Fig. 2A,C–D). This was also paralleled by a higher proliferation in *Trf1*^*Δ/Δ*^ *K5Cre Chk2*^*−/−*^ compared to *Trf1*^*Δ/Δ*^ *K5Cre Chk2*^*+/+*^ newborn epidermis as determined by Ki67-positive cells (Fig. 2A,E). Of note, despite this rescue, p53 levels were still significantly higher in *Trf1*^*Δ/Δ*^ *K5Cre Chk2*^*−/−*^ epidermis compared to wild-type, which may be indicative of p53 activation through the alternative ATR/Chk1 pathway (d’Adda di Fagagna *et al*., 2003; Zou & Elledge, 2003; Jazayeri *et al*., 2006; Martinez *et al*., 2009; Sfeir *et al*., 2009). In this regard, we observed a 3-fold increase in p-CHK1 levels in *Trf1*^*Δ/Δ*^ *K5Cre Chk2*^*−/−*^ compared to *Trf1*^*Δ/Δ*^ *K5Cre* keratinocytes (Fig. 2F).

### *Chk2* deficiency in *Trf1*^*Δ/Δ*^ *K5Cre Chk2*^*−/−*^ mice leads to epithelial abnormalities characteristic of telomere syndromes, including increased skin cancer

Interestingly, cell division in the presence of persistent DNA damage in long-lived *Trf1*^*Δ/Δ*^ *K5Cre Chk2*^*−/−*^ mice resulted in development of epithelial pathologies, which recapitulate some of the skin abnormalities characteristic of the human telomere syndromes, such as occurrence of nail dystrophy and fibropapilomas (Fig. 2G,H). In addition, the back skin displayed preneoplasic lesions such as epithelial hyperplasia and dysplasia, orthokeratotic hyperkeratosis, and dermal infiltrates. In addition, long-lived *Trf1*^*Δ/Δ*^ *K5Cre Chk2*^*−/−*^ mice also showed spontaneous development of invasive squamous cell carcinomas (SCC) both in the tail and ear skin (Fig. 2G,H).

Altogether, these results demonstrate that *Chk2* deletion attenuates induction of p53/p21 as well as rescues proliferative defects in the skin of *Trf1*^*Δ/Δ*^ *K5Cre* mice, which in turn leads to a partial rescue of survival but also to increased tumorigenesis.

### *Chk2* deficiency increased median survival of G1 and G2 *Terc*-deficient mice

**Figure 3:**
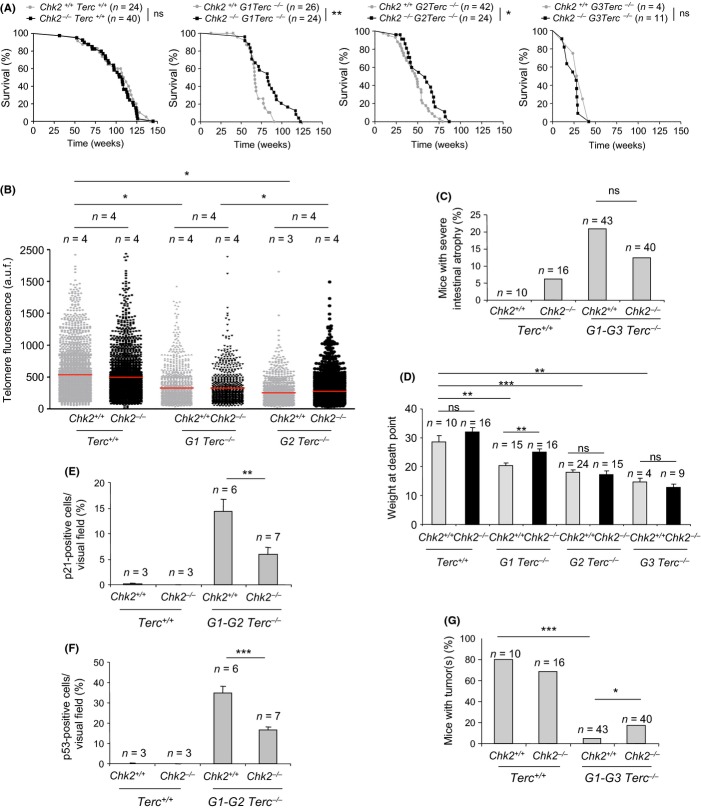
*Chk2* deficiency improves mouse survival, intestinal degenerative pathologies, and body weight of G1-G2 Terc-deficient mice (A) Survival curves of G1-G3 *Terc/Chk2* mouse cohorts. Statistical analysis was done by the Log-rank (Mantel–Cox) test. ns, no significant; *P < 0.05; **P < 0.01. (B) Telomere length analysis by q-FISH in the intestine of G1-G3 *Terc/Chk2* mouse cohorts. (C) Quantification of severe intestinal degenerative lesions in *Terc*^*+/+*^ and G1-G3 *Terc*^*−/−*^ in *Chk2*-proficient and deficient background at death point. (D) Body weight of G1-G3 Terc/Chk2 mouse cohorts at death point. (E–F) Percentage of p21-positive (E) and p53-positive cells (F) in the intestinal crypts of the indicated G1-G2 Terc/Chk2 mouse cohorts. (G) Percentage of mice that presented malignant tumors among G1-G3 *Terc/Chk2* cohorts at death point. *T*-Test was performed for statistical analysis in B, D, and E. Chi-square was performed for statistical analysis in F. Error bars represent standard error. *P < 0.05; **P < 0.01; ***P < 0.001. *n*, number of mice of each genotype.

To address the effect of *Chk2* deletion in a model of telomeric dysfunction originated by telomere shortening, we crossed *Chk2*^*−/−*^ with telomerase-deficient *Terc*^*−/−*^ knockout mice (Blasco *et al*., 1997; Hirao *et al*., 2002; Takai *et al*., 2002). The *Chk2*^*+/−*^ *Terc*^*+/−*^ mice were further crossed to generate successive generations (G1–G3) of *Chk2*^*−/−*^ *Terc*^*−/−*^ mice, which progressively shorten telomeres. Similar to the results obtained in *Trf1*^*Δ/Δ*^ MEFS, critically short telomeres induce Chk2 phosphorylation and the *Chk2*^*−/−*^ *G1-G3 Terc*^*−/−*^ presented similar incidence of chromosomal aberrations and senescent cells as the *Chk2*^*+/+*^ *G1-G3 Terc*^*−/−*^ MEFs (Fig. S2). Again, *in vivo* analysis of successive generations of these mice revealed that *Chk2* deletion could significantly rescue the survival of the first two generations (G1 and G2) of telomerase–deficient mice (Fig. 3A). This rescue was not due to an effect of Chk2 deletion on telomere length, as we detected similar telomere shortening in successive *of Chk2*^*−/−*^ *G1-G3 Terc*^*−/−*^ and *Chk2*^*+/+*^ *G1-G3 Terc*^*−/−*^ mice (Fig. 3B).

In line with the increased survival, *Chk2*^*−/−*^ *Terc*^*−/−*^ mice showed a reduced incidence of severe intestinal atrophy, a frequent cause of death in the *Terc*-deficient mice (Fig. 3C). Furthermore, decreased body weight presented by *Terc*^*−/−*^ mice at their time of death was rescued by *Chk2* deficiency in the first generation G1 *Terc*^*−/−*^ (Fig. 3D). At the molecular level, we observed a significantly decreased expression of p21 and p53 in intestinal crypts of *G1-G2 Chk2*^*−/−*^ *Terc*^*−/−*^ mice compared to the *Chk2*^*+/+*^ *G1-G2 Terc*^*−/−*^ controls (Fig. 3E,F), in line with the findings with *Chk2*^*−/−*^ *Trf1*^*−/−*^ mice (Fig. 2C,D). Of note, p53/p21 levels were still significantly higher in *G1-G2 Chk2*^*−/−*^ *Terc*^*−/−*^ intestines compared to wild-type, which indicates alternative mechanisms for p53/p21 activation (d’Adda di Fagagna *et al*., 2003; Zou & Elledge, 2003; Jazayeri *et al*., 2006; Martinez *et al*., 2009; Sfeir *et al*., 2009).

*Terc* deficiency has been previously shown to have a potent tumor suppressor effect with increasing mouse generations (Greenberg *et al*., 1998; Lee *et al*., 1998; Rudolph *et al*., 1999; Gonzalez-Suarez *et al*., 2000). In this regard, we found that, *Chk2* deletion lead to a slightly increased tumor incidence compared to the single *Terc*-deficient cohorts (Fig. 3G), suggesting that Chk2 could partially abolish the tumor suppressor effect of Terc deficiency.

## Discussion

Here, we studied the role of the checkpoint kinase *Chk2* in mediating the *in vivo* phenotypes induced by telomere dysfunction owing to either critical telomere shortening (*Terc* deficiency) or severe telomere uncapping (*Trf1* deficiency). We found that *Chk2* deficiency partially rescues both *Terc* deficiency in early generations and *Trf1* deficiency-associated phenotypes, thus demonstrating a role for Chk2 in signaling telomere dysfunction *in vivo*. Furthermore, we demonstrate that this rescue is not mediated by a significant rescue of the amount of DNA damage but instead reflects an attenuation of the proliferative defects associated to p53/p21 induction. A previous work reported that *Chk2* deletion in mice did not rescue survival or pathologies associated to *Terc* deficiency (Nalapareddy *et al*., 2010). Although our findings may seem to stand in contrast to those reported by Nalapareddy (Nalapareddy *et al*., 2010), it is relevant to note that these authors only focused their study in late generation iG4 telomerase-deficient mice which correspond to our G3 generation bearing very short telomeres and where we also do not see Chk2-mediated rescue. In contrast to our current study, Nalapareddy *et al*. did not address the effect of Chk2 deficiency in signaling critically short telomeres in G1 or G2 telomerase-deficient mice, which is where we observe a significant partial rescue in median survival by *Chk2* deficiency. In addition, the different outcomes might also be due to the different ways by which both groups have generated the *Terc*-deficient mouse cohorts. Thus, Nalapareddy *et al*. crossed G3 *Terc*^*−/−*^ *Chk2*^*+/−*^ with *Terc*^*+/−*^ *Chk2*^*+/−*^ to generate their experimental cohorts; namely, *Terc*^*+/−*^ *Chk2*^*+/+*^ (iF1 *Chk2*^*+/+*^), *Terc*^*+/−*^ *Chk2*^*−/−*^ (iF1 *Chk2*^*−/−*^), *Terc*^*−/−*^ *Chk2*^*+/+*^ (iG4 *Chk2*^*+/+*^), and *Terc*^*−/−*^ *Chk2*^*−/−*^ (iG4 *Chk2*^*+/+*^). The zygotes in all these cohorts harbor half of its chromosomes with long telomeres (those coming from the *Terc*^*+/−*^ parental) and half with short telomeres (those coming from the G3 *Terc*^*−/−*^ parental). In our work, we carried the *Terc*^*−*^ allele in homozygosis from the first generation of G1 *Terc*^*−/−*^ *Chk2*^*+/−*^ throughout the G2 and G3 generations, thus in a setting where all chromosomes present progressively shorten telomeres. It is conceivable that the Chk2-mediated response in G3 *Terc*-deficient mice is masked by a compensatory induction of a Chk2-independent mechanism that leads to p53/p21 activation. In fact, Nalapareddy *et al*. detected significantly higher levels of phosphorylated Chk1 in iG4 *Chk2*^*−/−*^ as compared to iG4 *Chk2*^*+/+*^ (Nalapareddy *et al*., 2010). In agreement with Nalapareddy *et al*., we also detected a slight increase in Chk1 phosphorylation in the absence of Chk2 in *Trf1*-deficient keratinocytes, which explains the fact that p53 levels were still elevated in *Chk2* cohorts compared to the wild-types. This fact also explains that *Chk2* deficiency only partially rescued survival and phenotypes associated to *Trf1* deficiency, while p53 deficiency was previously described by us to fully rescue *Trf1*^*Δ/Δ*^ *K5-Cre* perinatal lethality (Martinez *et al*., 2009). Thus, in the absence of Chk2, other components of the DDR, including Chk1 may be important to signal telomere dysfunction.

Finally, although single *Chk2* deficiency has not been associated to increased cancer (Donehower *et al*., 1992; Barlow *et al*., 1996; Hirao *et al*., 2002), we found a slightly increased cancer incidence in the absence of *Chk2* in the context of both *Trf1* and *Terc* deficiencies. These findings are in line, with increased cancer incidence associated to telomere dysfunction in the absence of p53. Interestingly, abrogation of other components of the DDR, such as *p21, Pms2 and Exo-1*, can rescue survival of *Terc*-deficient mice without increasing cancer (Choudhury *et al*., 2007; Schaetzlein *et al*., 2007; Siegl-Cachedenier *et al*., 2007).

In summary, our findings represent the first demonstration *in vivo* for a role of Chk2 in signaling telomere dysfunction owing to both short telomeres and severe telomere uncapping. Furthermore, we show a role for Chk2 in blocking tumorigenesis associated to dysfunctional telomeres.

## Supporting Information

**Fig. S1** *Chk2* deficiency does not rescue the Trf1-associated phenotypes in MEFs.

**Fig. S2** *Chk2* deficiency does not rescue *Terc*-associated phenotypes in MEFs.

**Data S1** Experimental procedures.
