# Supplementary material for: In vivo role of checkpoint kinase 2 in signaling telomere dysfunction
Source: Aging Cell. 2014 Jun 12;13(5):810–6. doi: 10.1111/acel.12237 (PMC4331747; doi:10.1111/acel.12237)
Supplement: Supplementary file 3 — Data S1 Experimental procedures. [file acel0013-0810-sd3.docx]

**Online supporting information**

**Experimental Procedures**

**Mice**

*Chk2^-/-^* (Hirao *et al.* 2002), *Terc^-/-^* (Blasco *et al.* 1997) and *Trf1^lox/lox^* *K5Cre^+/T^* (Martinez *et al.* 2009a) strains were inter-crossed to obtain *Terc^-/-^* *Chk2^-/-^* and *Trf1^Δ/Δ^ K5Cre Chk2^-/-^* mice. All mice were generated and maintained at the Spanish National Cancer Center under specific pathogen-free conditions in accordance with the recommendations of the Federation of European Laboratory Animal Science Associations (FELASA). All animal experiments were approved by the Ethical Committee and performed in accordance with the guidelines stated in the *International Guiding Principles for Biomedical Research Involving Animals*, developed by the Council for International Organizations of Medical Sciences (CIOMS).

**Cell Culture Assays**

MEFs were isolated from E13.5 embryos, expanded according to standard protocols, and were grown in DMEM supplemented with 10% fetal calf serum and antibiotics/ antimycotics. 1-3 × 10^6^ MEFs were plated in p150 plates and grown. 2 days before infection, they were put in serum starvation conditions (DNEM with 0,1% calf serum). When confluence was reached, MEFs were infected with adenoCre (4 x 10^6^ PFUs/plate, Iowa University -ILabsolutions) diluted in DNEM with 0,1% calf serum. After 48h of infection the medium was removed, new supplemented DNEM (10% serum) was added and assays were started. *Trf1* excision was monitored by PCR as previously described (Martinez *et al.* 2009a). For proliferation assays and β-galactosidase assay, 5 X 10^4^ infected MEFs were plated on six-well plates, with duplicates. Their growth rate was determined using an automatic cell counter (Millipore Scepter Automatic Cell Counter), on days 2, 3, 4 and 6 post-plating. β-galactosidase senescence-associated activity was detected using a commercial kit (Cell Signalling) on three time points (days 4; 6-7; 8-11 post-plating).

10000-30000 infected MEFs were plated in round coverslips for posterior fixation and immuno-FISH.

**Immuno-FISH**

Infected MEFs growing in round coverslips were fixed and subjected to immunofluorescence staining as described (Tarsounas *et al.* 2003), using anti-53BP1 (Ab-36823, Abcam). After washing, samples were fixed again in 4% formaldehyde in PBS, and telomeric FISH was performed as described previously (Samper *et al.* 2000), reducing formamide concentration to 30%.

**Q-FISH and cytogenetic analyses on metaphase spreads**

Infected MEFs were grown for 4 hours at 37°C in DMEM containing 1 μg/ml colcemid. After that, metaphases were prepared and FISH was performed as described previously (Samper *et al.* 2000).

At least 9 metaphases per genotype were analyzed for chromosomal aberrations by superimposing the Q-FISH telomere image on the DAPI image using Leica Q-FISH software. Cytogenetic analysis of metaphases was performed blindly as previously described (McNees *et al.* 2010).

**Western blotting**

Western blot was performed in keratinocytes and in MEFs following standard procedures. The antibodies used were raised against P-CHK1 (Ser 345, Cell Signaling), total CHK1 (G-4, sc-8408, Santa Cruz Biotechnology), CHK2 (Millipore 05-649).

**Histopathology and Immunohistochemistry**

For histopathological analysis, we isolated embryo skin, fixed it in 10% buffered formalin (Sigma) and embedded it in paraffin. The rest of the embryo was also fixed in formalin. Embryos were blindly studied by a pathologist, and all the stratified epithelia were analyzed (skin, oesophagus, tongue, cleft, nails, bladder).

Paraffin-embedded skin was sectioned and stained using the antibodies raised against: γ-H2AX Ser 139 (05-636, Millipore), 53BP1 (Ab-36823, Abcam), p21 (sc-397, Santa Cruz Biotechnology), p53 (1C12, Cell Signalling), Ki67 (Master Diagnostico), cytokeratin 6 (Covance).

**References**

Blasco MA, Lee HW, Hande MP, Samper E, Lansdorp PM, DePinho RA , Greider CW (1997). Telomere shortening and tumor formation by mouse cells lacking telomerase RNA. *Cell*. **91**, 25-34.

Hirao A, Cheung A, Duncan G, Girard PM, Elia AJ, Wakeham A, Okada H, Sarkissian T, Wong JA, Sakai T, De Stanchina E, Bristow RG, Suda T, Lowe SW, Jeggo PA, Elledge SJ , Mak TW (2002). Chk2 is a tumor suppressor that regulates apoptosis in both an ataxia telangiectasia mutated (ATM)-dependent and an ATM-independent manner. *Mol Cell Biol*. **22**, 6521-6532.

Martinez P, Thanasoula M, Munoz P, Liao C, Tejera A, McNees C, Flores JM, Fernandez-Capetillo O, Tarsounas M , Blasco MA (2009a). Increased telomere fragility and fusions resulting from TRF1 deficiency lead to degenerative pathologies and increased cancer in mice. *Genes Dev*. **23**, 2060-2075.

McNees CJ, Tejera AM, Martinez P, Murga M, Mulero F, Fernandez-Capetillo O , Blasco MA (2010). ATR suppresses telomere fragility and recombination but is dispensable for elongation of short telomeres by telomerase. *J Cell Biol*. **188**, 639-652.

Samper E, Goytisolo FA, Slijepcevic P, van Buul PP , Blasco MA (2000). Mammalian Ku86 protein prevents telomeric fusions independently of the length of TTAGGG repeats and the G-strand overhang. *EMBO Rep*. **1**, 244-252.

Tarsounas M, Davies D , West SC (2003). BRCA2-dependent and independent formation of RAD51 nuclear foci. *Oncogene*. **22**, 1115-1123.

**Supplementary Figure Legends**

**Supplementary Figure 1: *Chk2* deficiency does not rescue the Trf1-associated phenotypes in MEFs. (A)** *Trf1* deletion in MEFs upon Cre-mediated infection was confirmed by PCR amplification of *Trf1* locus. The different *Trf1^flox^* and *Trf1^Δ^* alleles are depicted. **(B)** Representative western blot images of phospho-CHK2 and CHK2 in the indicated genotypes. Actine was used as a loading control. **(C)** MEF growth curves of the indicated genotype. **(D)** Representative image of 53BP1 and telomeric PNA-probe immunoFISH performed on MEFs. Magnifications of foci are shown in the insets. Notice the co-localization of telomere and the 53BP1 foci found in *Trf1*-deleted MEFs. **(E)** Percentage of MEFs with three or more 53BP1 and γH2AX foci. **(F)** Number of telomeric induced foci (TIFs) per MEF, determined by 53BP1 and telomeric-probe immunoFISH. **(G)** Percentage of multitelomeric signals, chromosomal and chromatid fusions per metaphase. Representative image of the chromosomal aberrations are shown. **(H)** Percentage of senescent MEFs at days 4, 6-7, 8-11 post-infection, determined by β-galactosidase senescence-associated staining. T-Test was performed for statistical analysis. Error bars represent standard error. n=number of MEFs of each genotype. *, p<0.05; **, p<0.01; ***, p<0.001.

**Supplementary Figure 2: *Chk2* deficiency does not rescue *Terc*-associated phenotypes in MEFs. (A)** Representative western blot images of phospho-CHK2 and CHK2 in the indicated genotypes. Tubuline was used as a loading control. **(B-D)** Number of multitelomeric signals (MTS) (B), chromosome fusions (C) and chromatid fusion (D) events per metaphases of the indicated genotypes. **(D)** Percentage of senescent MEFs of the indicated genotypes. 500-800 cells were analyzed per genotype. T-test was performed for statistical analysis. Error bars represent standard error. The number of independent MEFs, of metaphases and of chromosomes analyzed in each case are indicated.
