# Supplementary material for: In vivo role of checkpoint kinase 2 in signaling telomere dysfunction
Source: Aging Cell. 2014 Jun 12;13(5):810–6. doi: 10.1111/acel.12237 (PMC4331747; doi:10.1111/acel.12237)

Supplementary Figure 2

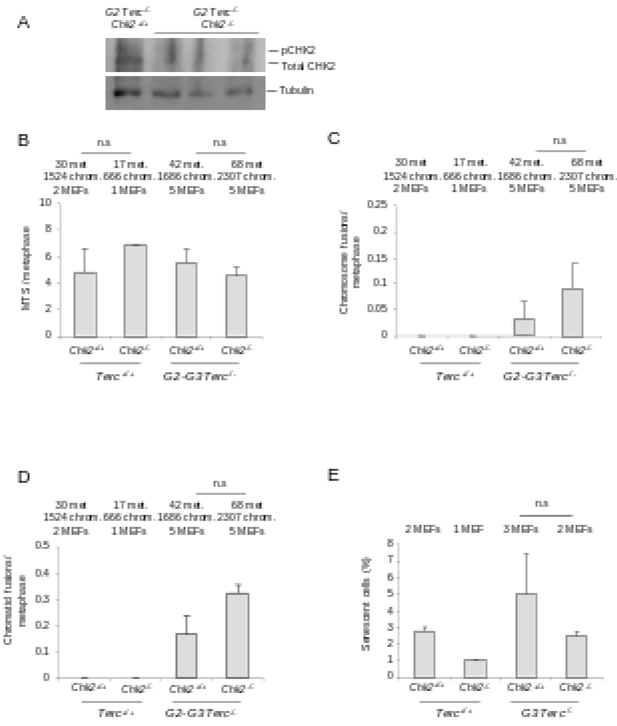

Supplement: Supplementary file 3 [file acel0013-0810-sd2.docx]
